# Socioeconomic factors associated with cessation of injection drug use among street-involved youth

**DOI:** 10.1186/s13011-017-0136-z

**Published:** 2017-12-07

**Authors:** Derek C. Chang, Scott E. Hadland, Ekaterina Nosova, Evan Wood, Thomas Kerr, Kora DeBeck

**Affiliations:** 1 British Columbia Centre on Substance Use, British Columbia Centre for Excellence in HIV/AIDS, St. Paul’s Hospital, 608-1081 Burrard Street, Vancouver, BC V6Z 1Y6 Canada; 2 Department of Family Medicine, University of British Columbia, St. Paul’s Hospital, 608-1081 Burrard Street, Vancouver, BC V6Z 1Y6 Canada; 3 Grayken Center for Addiction / Department of Pediatrics, Boston Medical Center, One Boston Medical Center Place, Boston, MA 02118 USA; 4 Division of General Pediatrics, Department of Pediatrics, Boston University School of Medicine, 88 East Newton St., Vose Hall, Room 322, Boston, MA 02118 USA; 5 Department of Medicine, University of British Columbia, St. Paul’s Hospital, 608-1081 Burrard Street, Vancouver, BC V6Z 1Y6 Canada; 6 Simon Fraser University, School of Public Policy, SFU Harbor Centre, 515 West Hastings Street, Suite 3271, Vancouver, BC V6B 5K3 Canada

**Keywords:** Youth, Injection drug, Cessation, Prohibited street-based income generation, Illegal income generation, Drug dealing

## Abstract

**Background:**

Although the initiation of injection drug use has been well characterized among at-risk youth, factors that support or impede cessation of injection drug use have received less attention. We sought to identify socioeconomic factors associated with cessation of injection drug use among street-involved youth.

**Methods:**

From September 2005 to May 2015, data were collected from the At-Risk Youth Study (ARYS), a prospective cohort study of street-involved youth in Vancouver, Canada. Multivariate extended Cox regression was utilized to identify socioeconomic factors associated with cessation of injection drug use for six months or longer among youth who were actively injecting.

**Results:**

Among 383 participants, 171 (44.6%) youth reported having ceased injection (crude incidence density 22 per 100 person-years; 95% confidence interval [CI], 19–26) at some point during study follow-up. Youth who had recently dealt drugs (adjusted hazard ration [AHR], 0.50; 95% CI, 0.29–0.87), engaged in prohibited street-based income generation (AHR, 0.41; 95% CI, 0.24–0.69), and engaged in illegal income generating activities (AHR, 0.19; 95% CI, 0.06–0.61) were significantly less likely to report cessation of injection drug use.

**Conclusions:**

Our findings suggest that socioeconomic factors, in particular engagement in prohibited street-based and illegal income generating activities, may pose barriers to ceasing injection drug use among this population. Effort to improve access to stable and secure income, as well as employment opportunities may assist youth in transitioning away from injection drug use.

**Trial registration:**

Our study is not a randomized controlled trial; thus the trial registration is not applicable.

## Background

Youth who are street-involved, defined as being homeless or using services for homeless youth, experience excess morbidity and mortality relative to the general population of adolescents and young adults [1, 2]. Although injection drug use is recognized as a risky activity by street-involved youth [2], it remains prevalent among this population and is associated with many harms, including infection with human immunodeficiency virus (HIV) and hepatitis C virus (HCV), as well as fatal overdose [1, 3, 4].

Among adult populations of people who inject drugs, numerous factors have been associated with cessation of injection drug use including stable housing, use of supervised injection facilities and engagement with addiction treatment [5, 6]. Among youth populations, multiple studies on drug use trajectories focus on the initiation of injection drug use, and point to the role of unemployment, homelessness, and inability to access addiction treatment as contributing factors to injection initiation [7–9]. Two longitudinal studies drawing on data from 1995 to 2000 and 2000–2008 respectively, found that homelessness, unemployment, and incarceration were associated with a lower likelihood of ceasing injecting among youth [10, 11]. Collectively, these findings suggest that economic vulnerability plays a role in drug use trajectories and may influence cessation of injection drug use. In an era of high rates of opioid overdose fatalities among young adults and adolescents who use drugs in North America [12, 13], updating the evidence base to better understand factors that influence drug use trajectories, specifically injection cessation, is particularly timely. Therefore, we sought to examine the potential relationship between socioeconomic factors and cessation of injection drug use among street-involved youth in Vancouver, Canada.

## Methods

The At-Risk Youth Study (ARYS) is an ongoing prospective cohort study of street-involved youth in Vancouver, Canada. This study has been described in detail previously [14]. In brief, snowball sampling and street-based outreach as well as self-referral were used to recruit participants into the study. Persons between 14 and 26 years of age who had used illicit drugs other than or in addition to cannabis in the past 30 days and provided informed consent were eligible to participate. At baseline and semiannually thereafter, participants complete an interviewer-administered questionnaire. The questionnaire elicits sociodemographic data as well as information regarding participants’ substance use and other behavioral and socioeconomic data such as housing, income sources, incarceration, and engagement with health and social services. All participants receive a monetary stipend of $30 (Canadian Dollar) after each interview. The University of British Columbia/Providence Health Care Research Ethics Board approved the study.

All participants who completed a baseline survey and were seen for study follow-up between September 2005 and May 2015 were eligible for the study. The present analysis was restricted to participants who reported active injection (i.e., those who reported any drug injection during the preceding six months, either at their baseline visit or at any follow-up visit) and who returned for at least one additional follow-up visit to assess for cessation of injection drug use. The primary outcome of interest was self-reported cessation of injection drug use during the preceding six months at any follow-up visit. Specifically, participants were asked, “In the last six months, have you used a needle to chip, fix, or muscle even once (yes vs. no)?”

Socioeconomic factors that we hypothesized might be associated with cessation of injection drug use included: homelessness; living with family; eviction from housing; living in the Downtown Eastside neighborhood (Vancouver’s drug use epicenter); employment (having a regular, temporary, or self-employed work); loss of income assistance (being cut off or denied income assistance); health care access (having been to a health care facility); incarceration (being in detention, prison or jail); sex work involvement (exchanging sex for money, gifts, or drugs); drug dealing; engaging in prohibited street-based income generating activities (panhandling, recycling, squeegeeing); and engaging in illegal income generating activities (theft, robbing, fraud, other illegal actives excluding sex work and drug dealing). All socioeconomic factors were time-updated measures based on activities or situations in the preceding six months time period. To protect against reverse causation whereby reported socioeconomic factors were a consequence of injection cessation, measures were taken from the study follow-up visit that preceded the visit at which a participant reported cessation of injection.

The following mental health related factors which we hypothesized might influence socioeconomic status were also considered: self-reported history of mental illness (defined as reporting having ever been diagnosed with a mental illness at study baseline); childhood physical or sexual abuse (defined as affirmative answers at study baseline to the question: “Have you ever been physically/ sexually abused?”); and depression at study baseline (based on the Center for Epidemiologic Studies Depression Scale >22). Mental health related measures were not time-updated.

We also considered sociodemographic and drug-use related factors that we hypothesized, based on a review of the prior available literature, might potentially confound the relationship between socioeconomic factors and injection cessation [10, 11]. These factors included: age (per year older); gender (female vs. male); ethnicity (Caucasian vs. non-Caucasian); high school completion; any heroin use; any prescription opioid use; any crystal meth use; any cocaine use and any crack use. As with the time-updated socioeconomic factors, measures for the drug-use variables were also lagged to the prior study visit. This allowed us to account for behaviors during the six months preceding injection cessation to avoid issues related to reverse causation whereby measures were a consequence of injection cessation and not predictors of cessation.

As a first step, we compared sociodemographic characteristics and socioeconomic factors between those who did and did not cease injection drug use at any time during follow-up using Pearson’s chi-squared test and Fisher’s exact test (for cell counts under 5) for categorical variables and the Wilcoxon test for continuous variables. Participants were right-censored at the time of their first cessation event (i.e.*,* no further person-time at risk was contributed by that participant), but if they reported resuming injection drug use at a later visit, they reentered the cohort of individuals at risk; participants who did not report any cessation were right-censored at the time of their last follow-up visit. We also used an extended Cox proportional hazards regression model with time-updated variables to examine bivariate associations between each of the sociodemographic and socioeconomic factors, and time to cessation of injection drug use. The extended Cox model has been validated [15] and widely used in previous studies [7, 16, 17]. The inclusion of time-updated covariates in an extended Cox model negates the requirement of the proportional hazards assumption [15]. Variables significant at *p* < 0.10 in bivariate analyses were eligible for inclusion in the final multivariate model, which used backward selection to identify the model with the best fit based on minimizing the Akaike Information Criterion (AIC). To help determine if our results were robust, we also ran a fixed multivariate model where all variables of interest were forced into a single model. In addition, multicollinearity was assessed in two ways. First, we assessed for multicollinearity at baseline using “ever ceased using drugs” as an outcome. We then applied variance inflation factors directly to the multivariable Cox model and used “injection drug use cessation” as an outcome. Analyses were performed using R version 3.2.4 (R Foundation for Statistical Computing, Vienna, Austria). All *p* values were two-sided and tests were considered significant at *p* < 0.05.

## Results

Overall, among 383 actively drug-injecting youth who returned for follow-up, the median age was 22 (interquartile range [IQR] 21–24) year, 248 (64.8%) were male and 276 (72.1%) were white. An additional 151 youth reported injection drug use at study enrollment but did not return or were not yet eligible to return for a study visit due to the nature of an open cohort study. The 383 youth who completed a study follow-up visit were similar to the 151 who did not with regard to all study variables at baseline (*p* > 0.05 for all), with the exception that individuals who did not complete a study follow-up visit were more likely to have begun using drugs at a younger age and inject cocaine. Participants contributed 765 person-years of total follow-up with a median of 19 months (IQR, 10–31) of follow-up per participant and a median of 3 (IQR, 2–5) study visits per participant. Based on the follow-up data, 171 (44.6%) youth reported cessation of injection drug use, resulting in a crude incidence density of 22 per 100 person-years (95% confidence interval [CI], 19–26 per 100 person-years).

Table 1 lists sociodemographic characteristics, drug use, mental health, and socioeconomic factors at baseline, stratified by injection cessation at any point during study follow-up. Youth did not differ according to sociodemographic and mental health characteristics at baseline. However, those who ceased injection over study follow-up were significantly more likely to have recently used heroin, prescription opioids, and accessed health care at baseline.

**Table 1:** Baseline characteristics^a^ of street youth who inject drugs stratified by whether they ceased injection at any point during study follow-up: At Risk Youth Study (ARYS), Vancouver, British Columbia, 2005–2015 (*n* = 383)

	Ceased Injection Drug Use^b^		
	Yes (%) (*n* = 171)	No (%) (*n* = 212)	*p* Value
Sociodemographic characteristics			
Median age, years (IQR)	22 (20–24)	22 (21–24)	0.769
Gender			
Male	103 (60.2)	144 (67.9)	0.118
Female	68 (39.8)	68 (32.1)	
Ethnicity			
Caucasian	126 (73.7)	150 (70.8)	0.573
Non-Caucasian	45 (26.3)	61 (28.8)	
High school education^c^			
Yes	59 (34.5)	62 (29.3)	0.281
No	111 (64.9)	148 (69.8)	
Drug use related factors			
Any heroin use			
Yes	114 (66.7)	175 (82.5)	<0.001
No	57 (33.3)	37 (17.5)	
Any prescription opioid use			
Yes	55 (32.2)	97 (45.8)	0.007
No	116 (67.8)	115 (54.2)	
Any crystal meth use			
Yes	116 (67.8)	162 (76.4)	0.061
No	55 (32.2)	50 (23.6)	
Any cocaine use			
Yes	78 (45.6)	97 (45.8)	0.978
No	93 (54.4)	115 (54.2)	
Any crack use			
Yes	113 (66.1)	131 (61.8)	0.385
No	58 (33.9)	81 (38.2)	
Mental health related factors			
Mental illness history			
Yes	104 (60.8)	138 (65.1)	0.388
No	67 (39.2)	74 (34.9)	
Childhood physical or sexual abuse			
Yes	116 (67.8)	142 (67.0)	0.933
No	45 (26.3)	54 (25.5)	
Depression			
Yes	75 (43.9)	105 (49.5)	0.057
No	53 (31)	46 (21.7)	
Socioeconomic factors			
Homeless			
Yes	121 (70.8)	160 (75.5)	0.305
No	49 (28.7)	51 (24.1)	
Living with family			
Yes	20 (11.7)	26 (12.3)	0.865
No	151 (88.3)	186 (87.7)	
Evicted			
Yes	18 (10.5)	25 (11.8)	0.664
No	82 (48.0)	132 (62.3)	
Living in the Downtown Eastside			
Yes	64 (37.4)	82 (38.7)	0.802
No	107 (62.6)	130 (61.3)	
Employed			
Yes	62 (36.3)	93 (43.9)	0.131
No	109 (63.7)	119 (56.1)	
Loss of income assistance			
Yes	13 (7.6)	18 (8.5)	0.899
No	108 (63.2)	157 (74.1)	
Accessed health care			
Yes	133 (77.8)	166 (78.3)	0.045
No	38 (22.2)	46 (21.7)	
Incarcerated			
Yes	35 (20.5)	46 (21.7)	0.792
No	135 (78.9)	166 (78.3)	
Sex work			
Yes	27 (15.8)	31 (14.6)	0.751
No	144 (84.2)	181 (85.4)	
Dealt drugs			
Yes	86 (50.3)	108 (50.9)	0.899
No	85 (49.7)	104 (49.1)	
Prohibited street-based income generating activities^d^			
Yes	54 (31.6)	82 (38.7)	0.149
No	117 (68.4)	130 (61.3)	
Illegal income generating activities^e^			
Yes	40 (23.4)	54 (25.5)	0.638
No	131 (76.6)	158 (74.5)	

^a^Characteristics reported at time of study enrollment

^b^Cells do not uniformly add up to column total due to missing values

^c^Prior completion of or current enrollment in high school

^d^Prohibited street-based income generating activities included panhandling, recycling, and squeegeeing

^e^Illegal income generating activities included theft, robbing, fraud, and other illegal actives excluding sex work and drug dealing

Table 2 displays unadjusted and adjusted hazard ratios for cessation of injection drug use and variables of interest. Adjusted models demonstrate that youth who had recently dealt drugs (AHR, 0.50; 95% CI, 0.29–0.87), engaged in prohibited street-based income generation (AHR, 0.41; 95% CI, 0.24–0.69), engaged in illegal income generating activities (AHR, 0.19; 95% CI, 0.06–0.61), or used heroin (AHR, 0.55; 95% CI, 0.34–0.87), were significantly less likely to report cessation of injection drug use. The results of the fixed multivariate model were all similar (data not shown) and no multicollinearity was detected based on aforementioned assessment.

**Table 2:** Unadjusted and adjusted hazard ratios (HR) for factors associated with cessation of injection drug use among street youth who inject drugs: At-Risk Youth Study (ARYS), Vancouver, British Columbia, 2005–2015 (*n* = 383)

	Unadjusted HR (95% CI)	Adjusted HR (95% CI)^a^	*p* Value^e^
Sociodemographic characteristics			
Age (per year older)	1.00 (0.99–1.01)		
Female Gender	1.01 (0.73–1.40)		
Caucasian Ethnicity	1.09 (0.75–1.60)		
High school education^b^	1.10 (0.79–1.54)		
Drug use related factors			
Any heroin use^c^	0.58 (0.42–0.81)	0.55 (0.34–0.87)	0.010
Any prescription opioid use^c^	0.75 (0.56–1.02)		
Any crystal meth use^c^	0.64 (0.47–0.88)	0.65 (0.42–1.01)	0.054
Any cocaine use^c^	1.16 (0.86–1.56)		
Any crack use^c^	1.08 (0.79–1.47)		
Mental health related factors			
Mental illness history	1.07 (0.76–1.54)		
Childhood physical or sexual abuse^d^	0.82 (0.56–1.18)		
Depression^d^	0.64 (0.43–0.93)	0.64 (0.41–1.01)	0.053
Socioeconomic factors			
Homeless^d^	0.68 (0.51–0.90)	1.25 (0.86–1.83)	0.246
Living with family^d^	1.76 (1.22–2.55)	1.21 (0.73–2.02)	0.459
Evicted^d^	0.44 (0.22–0.89)	0.59 (0.29–1.21)	0.152
Living in the Downtown Eastside^d^	0.58 (0.42–0.82)	0.67 (0.42–1.06)	0.085
Employed^d^	1.49 (1.11–2.01)		
Loss of income assistance^d^	0.54 (0.25–1.16)		
Accessed health care^d^	1.11 (0.81–1.54)		
Incarcerated^d^	0.57 (0.37–0.88)	0.83 (0.46–1.51)	0.546
Sex work^d^	0.40 (0.23–0.72)	0.62 (0.29–1.33)	0.221
Dealt drugs^d^	0.37 (0.25–0.56)	0.50 (0.29–0.87)	0.015
Prohibited street-based income generating activities^d,f^	0.50 (0.34–0.72)	0.41 (0.24–0.69)	0.001
Illegal income generating activities^d,g^	0.25 (0.13–0.50)	0.19 (0.06–0.61)	0.005

^a^Variables significant at *p* < 0.10 in bivariate models were eligible for possible inclusion in the multivariate model (extended Cox proportional hazards regression model); variables included in the final multivariate model were identified using a backward selection approach to minimize the Akaike Information Criterion (AIC)

^b^Denotes completion of or current enrollment in high school

^c^Includes both non-injection and injection use; drug use behaviors were lagged by one visit in order to assess behaviors during the 6 months when participants who ceased were still injecting

^d^Reported for the 6 months prior to the last follow-up visit at which a participant was still injecting

^e^ *P*-values refer to adjusted HR

^f^Prohibited street-based income generating activities included panhandling, recycling, and squeegeeing

^g^Illegal income generating activities included theft, robbing, fraud, and other illegal actives excluding sex work and drug dealing

## Discussion

In this prospective cohort of street-involved youth who inject drugs, 44 % of the participants reported having ceased injection drug use at some point during the study period. We found that recent engagement in drug dealing, prohibited street-based, and other illegal income generating activities may pose barriers to injection cessation among youth in our setting.

Our findings build on two previous studies of cessation of injection drug use among street-involved youth conducted by Steensma et al. in Montreal between 1995 and 2000 [10] and Evan et al. in San Francisco between 2000 and 2008 [11]. Similar to our study, both drew on data from a prospective cohort of young people who use illicit drugs. These studies found that homelessness, employment, and history of incarceration were negatively associated with cessation of injection drug use among street-involved youth. Although these specific variables were not found to be associated with injection cessation among our study sample, we did find that other markers of economic vulnerability, namely that generating income through unstable risky income sources correlated negatively with injection drug cessation.

Previous studies have demonstrated that street-involved youth are economically vulnerable and often resort to risky income generating activities including drug dealing (58%) and other prohibited and illegal street-based income sources (82%) [18, 19]. Youth who engaged in risky income generating activities are known to be at increased risk for homelessness, high intensity drug use, encounter with police, and violence [18]. Engaging in drug dealing is also known to be associated with markers of economic and social vulnerability including homelessness, crack cocaine use, and police violence [19].

Our study contributes to the understanding that stable and safe income sources are critical for the health and well-being of street-involved youth [18, 20, 21]. In particular, our findings suggest that stable income support could facilitate cessation of injection drug use in this population. This is consistent with the concept of “recovery capital” [22], which highlights the importance of internal and external resources to achieve and sustain cessation from risky substance use. Similarly, integrating youth into their communities is important for increasing their social capital and prospects for economic security [23]. Nonetheless, lack of meaningful employment and labor market exclusion still exist as barriers to employment for this population [18]. Previous studies have pointed to the need for targeted interventions to increase income security among street-involved youth. Proposed interventions include restructuring income assistance, providing low-threshold employment, and reducing barriers to traditional employment by addressing stigma and other harms of criminalization [18, 20, 24, 25]. The potential for these types of interventions to support injection cessation warrants further exploration.

There are several limitations to this study. First, the ARYS cohort is not a random sample. Our snowball sampling methods may have reduced heterogeneity and validity of the findings, although it is noteworthy that the characteristics of the ARYS sample are similar to those from other cohorts of street-involved youth [10, 11]. Another potential limitation of our sample is that participants who were lost to follow-up were more likely to have begun using drugs at an earlier age and more likely to report injecting cocaine. Both these characteristics are associated with higher risk activities [26] and therefore our study may overestimate the true occurrence of injection cessation among street-involved youth. Other potential limitations relate to the reliance on self-report for key measures of interest. Self-report may be affected by socially desirable responding and recall bias. The potential impacts could result to an over or under estimation of our outcome of interest though, overall, we expect the impacts to bias the results towards the null. Lastly, as with all observation studies, despite extensive adjustment for potential confounding, the independent associations that we observed could be influenced by other factors that we are unable to adjust for.

## Conclusions

In sum, our study suggests that economic vulnerability characterized by resorting to risky income generation strategies including drug dealing, prohibited street-based and other illegal activities, may pose barriers for street-involved youth to cease injection drug use. These findings underscore the potential for social interventions that provide stable and secured income sources to influence drug use trajectories and reduce drug related harm. Further study in this area is warranted.
